# A biomechanical research of foot pressure for lower extremity in gait wearing trail walking shoes

**DOI:** 10.1186/1757-1146-7-S1-A136

**Published:** 2014-04-08

**Authors:** Seung-Bum Park, Sae-Yeon Lee, Seong-Mi Kim, Yu-Jin Hwang, Kyoung-Youl Yoo

**Affiliations:** 1 Footwear Biomechanics Team, Footwear Industrial Promotion Center, Busan, Korea; 2 Busanil Science High School, Busan, Korea

## 

The aim of this study is to analyze foot pressure distribution of trail walking shoes while walking. Hiking, a recreational activity which is able to exercise whole body in the nature without any cost, has compositive effects which can reduce stress, strengthen muscles of entire body and improve cardiopulmonary function [1]. The Topography is hard near the surface and has rough characteristic because of rocks. These condition can lead to injuries to feet in hiking and aggravating fatigability of foot when people hike for a long time [2],so hiking boots which are specially functioned are encouraged because walking on the rough surface has latent dangerousness of injury [3]. Trail walking shoes generally provide more stability and support than regular walking shoes. Trail walking shoes are for natural trails. In rocky, rooted, dusty and muddy trails, a trail walking shoe gives added traction and support.

**Figure 1:**
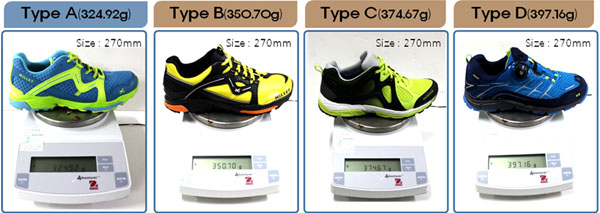
Type A: developed trail walking shoes, Type B: first developed trail walking shoes, Type C, D: other company’s trail wxalking shoes

Ten healthy males participated in this study. All subjects were free of lower extremity pain, history of serious injuries or operative treatment or subjective symptoms interfering with walking. Each subject wore four different shoe types during walking trials on a treadmill at a constant speed of 4.2km/hour. Pressure distribution data (contact area, maximum force, peak pressure, maximum mean pressure) were collected with pressure device at a sampling rate of 100Hz. Shoes used in the experiment are which developed in four shoes. Developed trail walking shoes (Type A), first developed trail walking shoes (Type B) and other company’s trail walking shoes (Type C, Type D) are selected for the experiment. Tested about ‘Comparison in Lightweightedness’ among the shoes (Figure 1). ‘Comparison in Lightweightedness’s result is Type A (324.92 g) < Type B (350.70 g) < Type C (374.67 g) < Type D (397.16 g).

**Table 1:** Result of Foot Pressure

Mask	Contact Area(cm^2^)					Maximum Force(N)				
	
	A	CA	B	C	D	A	CA	B	C	D
Total	142.187	142.739	142.049	146.076	139.403	677.276	703.008	708.275	715.217	690.800
M1	58.507	58.046	57.975	57.618	58.474	558.903	552.363	568.875	539.680	565.046
M2	42.356	44.444	42.660	47.957	40.930	152.873	194.191	168.769	215.201	173.182
M3	40.664	39.659	40.782	39.834	39.483	402.950	412.355	436.040	407.802	416.908

**Mask**	**Peak Pressure(kPa)**					**Maximum Mean Pressure(kPa)**				
	
	**A**	**CA**	**B**	**C**	**D**	**A**	**CA**	**B**	**C**	**D**

Total	237.516	272.143	256.418	273.346	270.940	78.084	79.048	79.767	76.533	81.563
M1	235.927	268.839	256.130	272.763	264.915	95.864	95.639	98.354	94.568	96.709
M2	87.566	111.221	98.818	126.280	96.161	44.034	50.660	47.934	53.151	48.168
M3	166.622	177.161	171.323	162.103	192.218	99.827	104.309	107.090	102.840	105.777

* **CA:** Control Average = (Type C + Type D)/2

Contact area of functional shoes (Type A) increased in comparison to general shoes (Type C, Type D). At the same time, foot pressure decreased in comparison to general shoes (Type C, Type D). It is expected that Type A Functional shoes give more comfort and fit by increasing the contact area and decreasing peak pressure.

In the result of the analysis of plantar pressure, Type A reported higher than other shoes on the forefoot. At the maximum force, Type A is observed as the smallest maximum force in almost part. These results, which is similar to Park (2009)’s research [3], decreased the confining pressure which can lead the deformation of forefoot’s toe. In the maximum pressure result, the outcome is similar to maximum force, which the smallest is Type A < Type D < Type B < Type C. This can decline the impulse which occurs in heel strike section, as a result, this can decrease the fatigability of foot in long-time walking. In addition, similar to Oh and Lee (2009)’s research [4], it can lighten the impulse force delivered to the body, as being the important factor which can decrease the weight to the leg joint. As examining the result of the average pressure, Type A < Type C < Type D < Type B is observed.

In this thesis, we analyzed the contact area of plantar pressure, maximum force, maximum pressure, average pressure. Through this result, we can know impact force alleviation for foot and physical fatigue, too.

When considering the pressure change of the foot, Type A’s contact area of foot is wider than the others. So, its wearing feeling will be better than the others. In case of maximum pressure, it is lower than the others and mid foot, hind foot’s result is similar. So, we expect ‘shockproof and to disperse pressure’ will be good. Also, with foot and shoe contact area’s increase, there may be amaximum force and maximum pressure decrease. So, it can decrease the foot’s and pelvic limb’s fatigue.

We offer the data of the dispersing pressure functionality of walking hiking shoes, so it can be of help to a product’s functionality improvement.
